# Sex Hormones and Keratoconus: In Search of the Link

**DOI:** 10.3390/jcm15041528

**Published:** 2026-02-14

**Authors:** Iasonas Makrypoulias, Irini Chatziralli, Dimitris Papaconstantinou, Konstantinos Panagiotopoulos, Stylianos A. Kandarakis, Petros Petrou, Anke Messerschmidt-Roth, Konstantinos Droutsas

**Affiliations:** 1 1st Department of Ophthalmology, General Hospital of Athens ‘Georgios Gennimatas’, National and Kapodistrian University of Athens, 11527 Athens, Greece; iasmakrip@med.uoa.gr (I.M.); dimpapac@med.uoa.gr (D.P.); s.kandarakis@gmail.com (S.A.K.); petroup@med.uoa.gr (P.P.); 2 2nd Department of Ophthalmology, Panepistemiako Geniko Nosokomeio Attikon, National and Kapodistrian University of Athens, 12462 Athens, Greece; eirchat@med.uoa.gr; 3 Biochemistry Laboratory, General Hospital of Athens ‘Georgios Gennimatas’, 11527 Athens, Greece; biochemical@gna-gennimatas.gr; 4 Department of Ophthalmology, University Hospital Marburg (UKGM), Philipps University of Marburg, 35037 Marburg, Germany; anke.messerschmidt-roth@med.uni-marburg.de

**Keywords:** keratoconus, sex hormones, corneal biomechanics

## Abstract

**Background**: Keratoconus (KC) is the most common ectatic corneal disorder, causing progressive corneal deformation, visual impairment, and reduced quality of life. Although KC pathogenesis is multifactorial, the contribution of systemic factors, including hormonal regulation, remains incompletely understood. This study aimed to investigate the role of sex hormones and gonadotropins in KC in a predominantly Greek population. **Methods**: We recruited 105 KC patients and 71 healthy controls (HC). Plasma levels of luteinizing hormone (LH), follicle-stimulating hormone (FSH), estradiol (E2), prolactin (PRL), testosterone (TES), dehydroepiandrosterone sulfate (DHEA-S), and progesterone (PRG) were measured and analyzed in relation to corneal tomographic and biomechanical parameters, as well as treatment modality. **Results**: LH showed positive correlations with corneal biomechanical parameters. KC patients who underwent penetrating keratoplasty exhibited higher FSH levels and a reduced LH/FSH ratio compared with those treated with corneal cross-linking. E2 levels were increased in women over 46 years of age, while PRL correlated with Kmax and Q-value. Men with KC demonstrated reduced TES associated with corneal morphology and biomechanics, increased PRG levels, and reduced DHEA-S in keratoplasty-treated patients. **Conclusions**: These findings suggest that sex hormones and gonadotropins may contribute to KC pathophysiology, supporting a systemic hormonal component in disease progression.

## 1. Introduction

Keratoconus (KC) is the most prevalent form of corneal ectasia and represents the leading entity within the classical triad of corneal ectatic disorders, which also includes keratoglobus and pellucid marginal corneal degeneration (PMD) [1]. Clinically, KC is characterized by stromal thinning accompanied by central or paracentral conical protrusion of the cornea which may progress, ultimately leading to irregular astigmatism, myopia, and a gradual decline in visual acuity [1,2,3].

The condition typically emerges during adolescence, affects asymmetrically both eyes, and progresses at variable rates until the third or fourth decade of life, when it usually stabilizes [4,5].

Epidemiological studies report considerable variation in the prevalence and incidence of KC across geographic regions. These discrepancies reflect not only genetic and environmental influences, such as increased exposure to ultraviolet radiation, but also differences in diagnostic methodologies. The prevalence of the disease has been increasing steadily for the last decades, most likely due to better diagnostic technologies [3,6,7]. In a recent meta-analysis, Hashemi et al. reported a KC prevalence of 1.38 per 1000 individuals in the general population, noting a slightly higher rate in men (20.6 per 1000) than in women (18.33 per 1000) among studies providing sex-specific data [8].

Despite extensive clinical and laboratory research, the etiopathogenesis of KC remains elusive, with genetic, environmental, and mechanical factors interacting through complex cellular and molecular mechanisms and leading to the appearance and progression of KC. Inflammation, keratocyte apoptosis, abnormal enzymatic activity, disturbances in collagen and proteoglycan synthesis, as well as altered corneal biomechanical stability have all been implicated, although the relative contribution of each remains unclear [9,10,11,12,13].

In recent years, increasing evidence suggests that hormonal imbalances constitute a critical yet previously underestimated component in the development of KC. Studies have identified notable changes in the levels of several sex hormones—including luteinizing hormone (LH), follicle stimulation hormone (FSH), prolactin (PRL), testosterone (TES), Dehydroepiandrosterone Sulfate (DHEA-S), and progesterone (PRG) estrone (E1), estradiol (E2) and estriole (E3)—in patients with KC when compared to their healthy counterparts [14,15,16,17,18,19,20]. Thyroid gland disorders have also been linked to KC [21]. Due to several limitation such as small sample sizes and variability among study populations these studies often report conflicting results. The presence and potential influence of sex hormone imbalances in the development and progression of KC remain unclear.

The primary aim of our study was to investigate potential imbalances in sex hormone levels in a Greek population with KC. Our goal was to provide new data and further insights in this complicated topic and also evaluate the potential use of these hormones as seromarkers for the diagnosis and management of the disease.

## 2. Materials and Methods

### 2.1. Study Design

In this cross-sectional cohort study, KC patients examined at the Cornea Service of the First Department of Ophthalmology, University Hospital of Athens “Georgios Gennimatas,” participated and were age-matched with healthy volunteers. Participants of Greek ethnicity underwent a comprehensive ophthalmologic evaluation, including refraction, slitlamp examination, Pentacam HR tomography, and Ocular Response Analyzer (ORA) assessment. These examinations were used to confirm the diagnosis of KC in affected individuals and to verify the absence of KC or other ocular pathology in healthy controls (HC). Kmax was used to classify patients into different severity groups. In addition, the same examination was performed after 6 months to assess disease progression based on clinical findings and Pentacam parameters (Kmax, TCT, BAD-D). Following ophthalmic assessment, venous blood samples were collected from all participants for the measurement of serum hormone levels, including LH, FSH, E2, PRL, TES, DHEA-S, and PRG.

### 2.2. Inclusion and Exclusion Criteria

Participants aged 16 years or older were eligible for the study. Individuals in the KC group were required to have a confirmed diagnosis of KC based on clinical examination and corneal tomography. HC were included only if they demonstrated no clinical signs of KC on slit-lamp examination and had normal corneal tomography with no evidence of ectatic disease or any other ocular pathology.

Exclusion criteria for both groups included a history of significant systemic illness, the use of hormonal contraceptives in female participants, the presence of other ocular diseases or corneal dystrophies, and any prior ocular surgery or trauma.

### 2.3. Sample Collection

Blood samples from all participants were collected in 10 mL tubes without anticoagulant. The tubes were then centrifuged at 4000 rpm for 15 min to obtain serum. The separated serum was transferred into sterile sample tubes and stored at −20 °C until subsequent analysis. All samples were collected in the morning between 8:00 and 11:00 a.m. to minimize potential systematic errors arising from circadian fluctuations in hormone levels [22,23]. Our initial plan for female participants was to collect samples exclusively during the luteal phase of their menstrual cycle to minimize hormonal variability. However, this proved exceedingly difficult due to the limited number of participants, challenges in scheduling follow-up visits, and irregular or unstable menstrual cycles in some individuals. Consequently, we adopted a more pragmatic approach by matching HC participants to the menstrual cycle phases of the corresponding KC patients, aiming to reduce variability while maintaining feasibility in sample collection.

### 2.4. Hormone Level Measurement

Serum levels of LH, FSH, E2, PRL, TES, DHEA-S, and PRG were measured using the in vitro chemiluminescent immunoassay method at the Maglumi 2000 analyser (Snibe Diagnostics, Shenzhen, China) [24] and at the Alinity Abbott analyser (Abbott Diagnostics, Abbott Park, IL, USA) [24].

For DHEA-S, a competitive chemiluminescence immunoassay was employed. The sample, buffer, magnetic microbeads coated with DHEA-S antigen and ABEI-labelled anti-DHEA-S antibody were thoroughly mixed and incubated. In this system, DHEA-S in the sample competes with the bead-bound antigen for binding to the labelled antibody, forming immunocomplexes. After magnetic separation, the supernatant was discarded and a wash cycle was performed. Starter 1 and Starter 2 were then added to trigger the chemiluminescent reaction. The emitted light, measured by a photomultiplier in relative light units (RLUs), is inversely proportional to the concentration of DHEA-S in the sample [24,25].

For the remaining hormones, a sandwich chemiluminescence immunoassay was used. The sample, buffer, and magnetic microbeads coated with a monoclonal antibody specific to each hormone were thoroughly mixed, incubated, and then subjected to magnetic separation followed by a wash cycle. A second monoclonal antibody, also specific for each hormone, was subsequently added, enabling the formation of sandwich complexes during incubation. After a second magnetic precipitation step, the supernatant was removed and another wash cycle was performed. A trigger solution was then added to initiate the chemiluminescent reaction. The emitted light, measured by a photomultiplier as relative light units (RLUs), is directly proportional to the concentration of each hormone in the sample [25,26].

All laboratory analyses were performed blindly, to minimize bias.

### 2.5. Ethics

This study was conducted in accordance with the principles of the Declaration of Helsinki. The study was approved by the National and Kapodistrian University of Athens (approval protocol number: 31785/3 April 2023) as well as the Review Board and Ethics committee the General Hospital of Athens “G. Gennimatas” (approval protocol number: 19385/19 July 2023). All participants signed a written informed consent before participation.

### 2.6. Statistical Analysis

To investigate the potential involvement of each hormone in KC, we performed a comprehensive series of non-parametric statistical analyses due to the non-normal distribution of our data. For every hormone measured, levels were first compared between KC patients and HC using the Mann–Whitney U test. To account for potential sex-related hormonal differences, all analyses were conducted separately in men and women, followed by age-stratified comparisons within each sex to account for possible age-related hormonal imbalances. For comparisons involving relatively small sample sizes in some analyses, the asymptotic *p*-value was not considered fully reliable. Therefore, the Monte Carlo method was additionally used to obtain more accurate probability estimates (exact significance), with the confidence interval set at 99% (N of hypothetical samples = 10,000). To explore potential associations between hormonal status and disease behavior, hormone levels were also compared between KC patients exhibiting Pentacam documented progression over a 6-month period and those without progression. Furthermore, Spearman’s correlation analyses were conducted for each hormone to assess associations with morphological parameters obtained from the Pentacam [Kmax, thinnest corneal thickness (TCT), central corneal thickness (CCT), Q-val(f), Q-val(b), front elevation (Elev(f)), back elevation (Elev(b))] and biomechanical parameters obtained from the ORA (corneal hysteresis (CH), corneal resistance factor (CRF)], separately in men and women. In addition, KC patients were categorized according to previous surgical interventions [no treatment, corneal cross-linking (CXL), penetrating keratoplasty (PK)), and hormone levels were compared across these groups using the Kruskal–Wallis H test. Lastly, a multiple linear regression analysis was performed in the whole cohort to examine whether plasma levels of each hormone were predictive of Pentacam and ORA parameters.

All analyses were performed using IBM SPSS^®^ Statistics v29 for Windows. The confidence level was set at 95% and the significance threshold at 5%.

## 3. Results

In total, 105 patients with KC and 71 HC were initially included in the study. One male patient in the KC group was found to have previously undiagnosed cryptorchidism and was therefore excluded, resulting in a final study population of 104 KC patients (84 males and 20 females) and 71 HC (51 males and 20 females).

Table 1 summarizes the population characteristics, including sex distribution, age group stratification, menstrual cycle phase among female participants, treatment status, disease progression at 6 months and disease severity in the KC group.

### 3.1. Hormone Levels in KC vs. HC

Table 2 summarizes the median serum hormone levels in patients with KC and HC. Median concentrations of LH, FSH, E2, PRL, TES, DHEAS, and PRG, as well as the LH/FSH ratio, were comparable between the two groups. Overall, no statistically significant differences were observed in serum hormone levels between patients with KC and HC (all *p* > 0.05).

### 3.2. Sex-Specific Differences in Serum Hormone Levels

Table 3 presents sex-specific comparisons of serum hormone levels between patients with KC and HC. Among male participants, TES and PRG levels differed significantly between groups, with lower TES and higher PRG observed in patients with KC compared with HC (*p* = 0.007 and *p* = 0.028, respectively). No statistically significant differences were detected in LH, FSH, the LH/FSH ratio, E2, PRL, or DHEAS between male KC patients and controls. Among female participants, serum levels of all evaluated hormones were comparable between patients with KC and HC, with no statistically significant differences observed (all *p* > 0.05).

### 3.3. Age Dependence

Age-stratified hormonal profiles were evaluated between patients with KC and HC in both men and women.

Among male participants aged 16–30 years, TES levels were significantly lower in patients with KC compared with HC (median 4.89 vs. 6.03 ng/mL, *p* = 0.020). In the 31–45-year age group, progesterone levels were significantly higher in male KC than in controls (median 0.20 vs. 0.10 ng/mL, *p* = 0.013). No statistically significant differences were observed for LH, FSH, LH/FSH ratio, E2, PRL, or DHEAS across age categories (Table 4).

In the female subgroup, the Monte Carlo method was additionally applied with a 99% confidence interval. E2 plasma levels were increased in KC patients older than 45 years compared with HC (median 48.45 (34–72.1) vs. 35.00 (34–37) pmol/L; Monte Carlo *p* = 0.029), whereas no statistically significant differences were observed for the remaining hormones across age groups (Table 5).

### 3.4. KC with Progression vs. KC Without Progression over a 6 Months Period

In addition, KC patients were categorized into two groups based on whether they demonstrated disease progression on Pentacam over a 6-month period, in order to assess whether hormonal imbalances played a role. The Monte Carlo method was also used due to small sample size. No significant correlations were found (Table 6).

### 3.5. Hormonal Levels in KC Based on Treatment Method

In a separate analysis, KC patients were grouped according to the medical procedures they had undergone. Three categories were created: patients with no surgical intervention, those who underwent corneal cross-linking (CXL), and those who underwent penetrating keratoplasty (PK). The Kruskal–Wallis H test was used and the Monte Carlo method was also applied, due to small sample sizes. When statistically significant differences were identified, post hoc analyses were performed to determine between which groups the significant differences occurred. Post hoc comparisons revealed that KC patients who underwent PK had higher plasma FSH levels than those who underwent CXL as well as a lower LH/FSH ratio. In addition, KC patients who underwent PK had a lower DHEAS levels compared to those who underwent CXL and lower plasma PRG levels (Table 7).

### 3.6. Associations Between Pentacam/ORA Values and Hormone Levels

Spearman’s correlation analysis was conducted to evaluate the associations between Pentacam and ORA parameters (Kmax, TCT, CCT, Q-val(f), Q-val(b), Elev(f), Elev(b), CH, CRF) and each hormone. The analysis was performed separately for men and women.

In men we observed a positive correlation of PRL with Kmax and a negative correlation with Q-val(f). In addition, plasma TES levels in men showed a negative correlation with Kmax, a positive correlation with TCT, a positive correlation with CCT, a negative correlation with Elev(f), a negative correlation with Elev(b) and a positive correlation with CH. Plasma PRG levels in men showed a negative correlation with TCT and a negative correlation with CCT (Table 8).

The same analysis was performed for women but no significant associations were found.

### 3.7. Linear Regression Analysis of Hormonal Predictors of Pentacam and ORA Parameters

A linear regression analysis was performed in the whole cohort to examine whether plasma levels of each hormone, as well as the LH/FSH ratio, were predictive of Pentacam and ORA. Among all hormones examined, only LH yielded statistically significant regression models, predicting CH (F(1,173) = 4.79, *p* = 0.036, R^2^ = 0.025) and CRF (F(1,173) = 5.89, *p* = 0.016, R^2^ = 0.033). Specifically, LH emerged as a positive predictor for both CH (B = 0.031, SE = 0.014, β = 0.158, *p* = 0.036) and CRF (B = 0.056, SE = 0.023, β = 0.181, *p* = 0.016). None of the remaining hormones or the LH/FSH ratio included in the models demonstrated significant predictive value for Pentacam or ORA parameters (Table 9).

### 3.8. Associations Between Disease Severity and Hormone Levels

Table 10 presents a comparison of hormone levels among patients with KC, categorized according to Kmax values. The patients were stratified into four groups: <49 D, 49–53 D, 53.1–58 D, and ≥58 D. Statistical analysis was performed using the Kruskal–Wallis H test, with the Monte Carlo method applied to account for small sample sizes. No statistically significant correlations were identified among the groups.

## 4. Discussion

For many years, the human eye was regarded as largely unaffected by sex-related factors. It is now clear, however, that sex significantly influence ocular physiology and the predisposition to develop certain diseases. One of the earliest documented connections between sex hormones and eye disease dates back to 1930, when Henrik Sjögren noted that hormonal fluctuations in women were linked to the development of dry eye syndrome [27]. In KC, a hormonal influence has long been hypothesized, supported by its tendency to emerge around puberty [28], to progress during pregnancy [29,30,31,32], and to show signs of stabilization after menopause [32,33,34]. Moreover, the use of exogenous hormones, such as those in contraceptives, hormonal stimulation for in vitro fertilization, and hormone replacement therapy, has also been associated with various corneal alterations [35,36,37,38,39]. The findings of studies investigating a potential sex predominance in KC are conflicting. Some studies report that KC occurs more frequently in women (53% to 66%) [40,41], while others report a male predominance [8,42,43] or find no significant difference between the sexes [44]. Overall, there appears to be a slight predominance in men; however, it is too small to draw a definitive conclusion about whether KC occurs more often in men or women [3].

Sex hormones are distributed throughout the body via the bloodstream, influencing a wide range of organs and tissues. Their physiological impact is largely determined by whether the cells of the targeted organs express the appropriate hormone receptors. In humans, multiple studies have demonstrated the presence of estrogen [45,46,47,48,49], androgen [45,48,50] LH [20,51], FSH [20,51], and progesterone [47,48,49] receptors across various ocular tissues, including the cornea. Moreover, emerging evidence suggests that the expression of these receptors may be altered in the corneas of patients with KC [47,52]. Sex hormones have also been detected in tear fluid [53,54,55]. Given the continuous interaction between tears and the ocular surface, fluctuations in tear hormone levels may influence corneal homeostasis.

Recent studies have investigated the relationship between KC and the levels of sex hormones. Zhao et al. reported higher plasma E2 levels in male KC patients compared to controls, with a weak positive correlation between E2 and Kmax. They also reported lower levels of testosterone in men and women plasma and a non-significant difference in the levels of plasma progesterone [14]. McKay T.B et al. performed salivary analyses in patients with KC and a control group, revealing that there was significant downregulation of estrone, in the female population with KC, and upregulation of DHEAS in the male KC population, respectively [15]. They found no significant changes in E2 levels [15]. Van L’s group compared the levels of sex hormones in KC patients before and 2–3 months after they underwent a CXL procedure and reported a positive correlation of E3 with Kmax, whereas DHEAS and E1 levels showed no significant association with either Kmax or CCTmin [16]. Sharif R et al. reported an upregulation of E1,E3 and DHEAS in the plasma and saliva of KC patients while the measured no difference in the levels of E2 between the KC and the control groups [17]. Jamali H’s team aimed to evaluate serum LH, FSH, androgen and PRL levels in KC patients and reported no significant alternations in the levels of FSH and LH, while an upregulation of PRL, DHEAS and TES was found [18]. In a different study Stachon T et al. reported a significant downregulation of PRL in the aqueous humor of patients with KC when compared to their healthy counter parts [19]. Karamichos D et al. investigated the role of gonadotropins in KC and found no significant changes in LH or FSH levels. However, they reported a significant reduction in the LH/FSH ratio in the plasma of KC patients [20].

Our study aimed to expand the current understanding of hormonal involvement in KC by evaluating, for the first time, all major sex hormones and gonadotropins simultaneously within the same patient group. We believe that this comprehensive approach allows the assessment of hormone–hormone interactions and reduces the risk of inconsistencies that may arise when studies examine isolated hormones in separate populations. Our findings partially align with and partially diverge from previous research. LH was a positive predictor of both CH and CRF. In addition, KC patients who underwent PK exhibited higher plasma FSH levels and a lower LH/FSH ratio compared with those who underwent CXL. These findings are in agreement with, and further extend, the results reported by Karamichos et al. [20]. Regarding E2 our results partially coincide with Zhao et al. team [14]. Taking into consideration our relatively few women participants, we hypothesize that the observed upregulation of E2 in women over 46 years of age may reflect a broader imbalance of E2 throughout life, which becomes detectable only after menopause, when the cyclical fluctuations of the menstrual cycle are no longer present. Our PRL findings are consistent with those of Jamali’s team [18], as we also observed a positive correlation between plasma PRL levels and Kmax. Additionally, we identified a negative correlation with Q-value, suggesting a strong likelihood that PRL plays a role in KC development. Concerning androgens we found a significant downregulation of TES in men with KC, which, together with its correlations with corneal morphology and biomechanics, supports earlier findings by Zhao et al. [14]. By integrating both Pentacam and ORA parameters, our results provide insight into the potential impact of low TES levels early in life on the development and progression of KC. We believe this difference is more pronounced in younger men, as testosterone naturally declines with age. Additionally, we found reduced DHEAS levels in KC patients who underwent PK compared to those who underwent CXL. Collectively, these findings suggest that early-life downregulation of androgens may play a critical role in both the development and progression of KC, while also diverging from the results reported by Jamali et al. [18]. Finally, we observed an upregulation of PRG in men with KC, representing a notable deviation from existing literature, which generally reports no significant changes in this hormone. Additionally, KC patients who underwent PK exhibited lower PRG levels compared to those who underwent CXL, suggesting a complex relationship between PRG levels and KC pathogenesis.

The molecular mechanisms linking sex hormones to KC progression remain unclear, but growing evidence suggests that hormonal influence on matrix metalloproteinases (MMPs) may play a central role. MMPs are zinc-dependent enzymes responsible for degrading collagen and other extracellular matrix (ECM) components. A dysregulation of their activity can weaken corneal structure over time [56,57,58]. Elevated PRL levels have been shown to reduced interleukin 6 (IL-6) and interleukin 8 (IL-8) secretion in KC corneal stromal fibroblasts in vitro [59] suggesting an interaction between PRL and cytokine regulation in the context of KC. Yin et al. found that E2 treatment reduced both mRNA and protein levels of MMP-2 in human corneal stromal cells, indicating a potential protective effect [60]. In contrast, Suzuki et al. demonstrated that E2 increased proinflammatory cytokines and upregulated multiple MMPs (MMP-2, -7, and -9) in corneal epithelial cells [61]. Escandon et al. used a 3D in vitro corneal model and demonstrated that healthy stromal cells (HCFs) and KC stromal cells (HKCs) exhibit distinct responses when exposed to different concentrations of E1 and E3. Their results indicate that these hormones influence the regulation of estrogen, androgen, and PRG receptors of the human cornea [46]. Together, these findings underscore the complex and cell-specific effects of sex hormones on inflammatory signaling and ECM remodeling in KC.

In addition, a number of biomarkers have been investigated in relation to KC [62], with Prolactin-Induced Protein (PIP) being one of the proposed candidates [17]. Studies have found that PIP levels are lower in the tears, saliva, and plasma of KC patients compared with healthy individuals [17,63], indicating its possible role in early diagnosis and monitoring disease progression. Furthermore, PIP expression appears to increase in stromal cells from KC donors following corneal collagen cross-linking (CXL) [64]. From a molecular point of view, reduced PIP expression in KC is linked to impaired ATP production from both oxidative phosphorylation and glycolysis, reflecting a state of metabolic insufficiency [65]. Restoration of PIP activity enhances mitochondrial respiration and glycolytic flux in keratoconic cells, indicating that PIP plays a key role in maintaining corneal stromal bioenergetic homeostasis [65]. Taken together, these emerging findings, together with the sex hormone regulation of PIP [66] and the associations between circulating sex hormones and CT and biomechanical parameters observed in our study, suggest that hormonally mediated mechanisms may contribute to KC onset and progression.

Overall, an accumulating body of evidence indicates that altered sex hormone activity and dysregulation of their receptors may play an important role in the development of KC. These findings further support the idea that persistent hormonal abnormalities, potentially present from early life, could contribute to initiating the disease. Our research shares several limitations common to previous studies in this field. Small sample sizes, resulting in reduced reliability and limiting the generalizability of the findings, low patient turnover, genetic variability even within a population of the same ethnicity, and environmental factors introduce unavoidable heterogeneity. In addition, the bioavailability of sex hormones spans a wide physiological range, particularly in female participants due to menstrual cycle variations, and fluctuates throughout the day further complicating the isolation of their specific contribution to KC. Furthermore, due to the cross-sectional design and single time-point hormone measurements, causality or directionality cannot be determined, and our findings should be interpreted only as associations.

## 5. Conclusions

Despite these constraints, our study provides meaningful insight into the complex relationship between sex hormones, gonadotropins, and KC, contributing novel evidence to an area that remains incompletely understood. By evaluating all major sex hormones and gonadotropins simultaneously within the same patient group, this work offers reduces inconsistencies inherent to studies focusing on isolated hormones. The observed associations between specific hormonal profiles and corneal tomographic and biomechanical parameters, as well as differences related to disease severity, support the concept of a systemic hormonal component in KC pathophysiology. Collectively, our findings confirm the complex and multifaceted relationship between hormonal regulation and KC, and indicate that future studies exploring a broader and more comprehensive panel of hormones are warranted to enable further discoveries and robust biomarker identification for KC susceptibility, disease progression, and clinical stratification.

## Figures and Tables

**Table 1:** Population Characteristics.

Sub-Groups	Keratoconus (n = 104)	Healthy Controls (n = 71)
**Gender, n (%)**		
Male	84 (80.77)	51 (71.83)
Female	20 (19.23)	20 (28.17)
**Age groups, n (%)**		
16–30 years	50 (48.08)	34 (47.89)
31–45 years	28 (26.92)	20 (28.17)
≥46 years	26 (25.00)	17 (23.94)
**Menstrual cycle phase, n (%)**	(n = 20)	(n = 20)
Follicular phase	3 (15.0)	3 (15.0)
Ovulation	4 (20.0)	4 (20.0)
Luteal phase	7 (35.0)	7 (35.0)
Menopause	6 (30.0)	6 (30.0)
**Treatment, n (%)**		
Corneal collagen cross-linking (CXL)	26 (25.00)	-
Penetrating keratoplasty (PK)	11 (10.58)	-
No surgical treatment	67 (64.42)	-
**Progression at 6 months, n (%)**		
Progression	12 (11.54)	-
Medical procedure	2 (1.92)	-
No progression	63 (60.57)	-
No data	27 (25.96)	-
**Severity, n (%)**		
Kmax: <49 D	8 (7.69)	-
Kmax: 49–53 D	11 (10.58)	-
Kmax: 53.1–58 D	28 (26.93)	-
Kmax >58 D	57 (54.80)	-

**Table 2:** Serum hormone levels in patients with KC vs. HC.

Hormone	KC (n = 104)	HC (n = 71)	*p* Value
LH (mIU/mL), median	3.43 (0.91–55.78)	3.16 (1.01–49.63)	0.950
FSH (mIU/mL), median	3.57 (0.56–79.36)	4.03 (0.74–81.54)	0.620
LH/FSH ratio, median	0.85 (0.21–9.54)	0.96 (0.22–3.68)	0.853
E2 (pmol/L), median	108.85 (34–784.9)	109.40 (34–1168.3)	0.727
PRL (ng/mL), median	9.89 (3.2–51.78)	8.25 (3.91–25.45)	0.353
TES (ng/mL), median	3.99 (0.2–12.11)	5.03 (0.17–10.27)	0.393
DHEAS (μg/dL), median	234 (25–747)	230 (35–529)	0.639
PRG (ng/mL), median	0.20 (0.1–13.8)	0.20 (0.1–18.7)	0.218

Abbreviations: LH, luteinizing hormone; FSH, follicle-stimulating hormone; E2, estradiol; PRL, prolactin; TES, testosterone; DHEAS, dehydroepiandrosterone sulfate; PRG, progesterone; KC, Keratoconus; HC, Healthy Controls.

**Table 3:** Sex-specific hormonal profiles of patients with KC and HC.

Hormone	Sex	KC	HC	*p* Value
LH (mIU/mL), median	Male (n = 84/51)	3.07 (0.91–8.35)	2.87 (1.01–11.17)	0.537
	Female (n = 20/20)	8.75 (2.26–55.78)	7.33 (1.8–49.63)	0.766
FSH (mIU/mL), median	Male (n = 84/51)	3.25 (0.56–23.14)	3.21 (0.74–24.18)	0.915
	Female (n = 20/20)	5.97 (2.44–79.36)	5.72 (2.08–81.54)	0.808
LH/FSH ratio, median	Male (n = 84/51)	0.82 (0.29–9.54)	1.00 (0.22–3.68)	0.599
	Female (n = 20/20)	0.97 (0.21–3.01)	0.91 (0.25–2.92)	0.387
E2 (pmol/L), median	Male (n = 84/51)	104.25 (34–231)	96.80 (34–231)	0.284
	Female (n = 20/20)	198.30 (34–784.9)	286.80 (34–1168.3)	0.786
PRL (ng/mL), median	Male (n = 84/51)	9.85 (3.2–51.78)	7.55 (3.91–21.84)	0.106
	Female (n = 20/20)	11.43 (4.5–31.55)	11.48 (4.45–25.45)	0.871
TES (ng/mL), median	Male (n = 84/51)	4.86 (1.45–12.11)	5.85 (0.89–10.27)	**0.007**
	Female (n = 20/20)	0.36 (0.2–0.82)	0.36 (0.17–0.77)	0.725
DHEAS (µg/dL), median	Male (n = 84/51)	275.00 (25–747)	252.00 (35–529)	0.506
	Female (n = 20/20)	152.00 (29–413)	192.50 (44–487)	0.402
PRG (ng/mL), median	Male (n = 84/51)	0.20 (0.1–0.6)	0.10 (0.1–0.6)	**0.028**
	Female (n = 20/20)	0.30 (0.1–13.8)	0.40 (0.1–18.7)	0.880

Abbreviations: LH, luteinizing hormone; FSH, follicle-stimulating hormone; E2, estradiol; PRL, prolactin; TES, testosterone; DHEAS, dehydroepiandrosterone sulfate; PRG, progesterone; KC, Keratoconus; HC, Healthy Controls. Values in bold indicate statistical significance.

**Table 4:** Age dependent hormonal profiles of male patients with KC and HC.

Hormone	Age	KC	HC	*p* Value
LH (mIU/mL), median	16–30 (n = 44/29)	3.24 (0.91–6.05)	2.68 (1.03–10.05)	0.183
	31–45 (n = 20/11)	2.56 (1.31–6.43)	3.34 (1.44–5.33)	0.375
	>45 (n = 20/11)	4.17 (1.79–8.35)	3.22 (1.01–11.47)	0.836
FSH (mIU/mL), median	16–30 (n = 44/29)	2.93 (0.56–8.83)	2.64 (0.74–9.26)	1.000
	31–45 (n = 20/11)	3.20 (1.38–7.51)	3.21 (1.53–5.79)	0.421
	>45 (n = 20/11)	6.60 (2.31–23.14)	4.58 (2.13–24.18)	0.433
LH/FSH ratio, median	16–30 (n = 44/29)	1.02 (0.31–9.54)	1.05 (0.32–3.68)	0.795
	31–45 (n = 20/11)	0.83 (0.38–1.99)	1.00 (0.29–1.89)	0.710
	>45 (n = 20/11)	0.50 (0.29–1.94)	0.73 (0.22–1.12)	0.180
E2 (pmol/L), median	16–30 (n = 44/29)	108.85 (34–231)	103.70 (34–202.4)	0.620
	31–45 (n = 20/11)	112.75 (34–212.3)	76.30 (34–123.7)	0.086
	>45 (n = 20/11)	99.15 (34–203.5)	93.30 (34–175)	0.918
PRL (ng/mL), median	16–30 (n = 44/29)	10.43 (4.14–51.78)	8.24 (4.12–16.04)	0.131
	31–45 (n = 20/11)	9.25 (4.35–17.52)	7.35 (3.91–21.84)	0.577
	>45 (n = 20/11)	9.13 (3.2–13.95)	7.46 (5.0–13.28)	0.433
TES (ng/mL), median	16–30 (n = 44/29)	4.89 (1.45–10.7)	6.03 (0.89–10.27)	**0.020**
	31–45 (n = 20/11)	3.97 (2.51–12.11)	5.38 (3.97–9.23)	0.052
	>45 (n = 20/11)	5.09 (2.3–8.49)	5.78 (2.95–8.03)	0.680
DHEAS (µg/dL), median	16–30 (n = 43/29)	314.0 (184–747)	293.0 (104–529)	0.249
	31–45 (n = 20/11)	276.0 (104–448)	252.0 (92–348)	0.215
	>45 (n = 20/11)	109.0 (24–336)	172.0 (35–425)	0.160
PRG (ng/mL), median	16–30 (n = 44/29)	0.20 (0.1–0.5)	0.20 (0.1–0.6)	0.095
	31–45 (n = 20/11)	0.20 (0.1–0.3)	0.10 (0.1–0.2)	**0.013**
	>45 (n = 20/11)	0.10 (0.1–0.6)	0.10 (0.1–0.3)	0.713

Abbreviations: LH, luteinizing hormone; FSH, follicle-stimulating hormone; E2, estradiol; PRL, prolactin; TES, testosterone; DHEAS, dehydroepiandrosterone sulfate; PRG, progesterone; KC, Keratoconus; HC, Healthy Controls. Values in bold indicate statistical significance.

**Table 5:** Age dependent hormonal profiles of female patients with KC and HC.

Hormone	Age	KC	HC	*p*-Value/Monte Carlo *p*-Value
LH (mIU/mL), median	16–30 (n = 6/6)	7.11 (4.52–55.78)	6.80 (1.8–20.49)	0.631/0.702
	31–45 (n = 8/8)	5.40 (2.26–20.96)	4.41 (3.08–22.74)	0.916/0.960
	>45 (n = 6/6)	23.08 (15.59–31.69)	27.07 (15.76–49.63)	522/0.588
FSH (mIU/mL), median	16–30 (n = 6/6)	5.97 (2.6–19.47)	4.58 (2.38–7.01)	0.337/0.398
	31–45 (n = 8/8)	4.10 (2.44–6.96)	4.55 (2.08–23.85)	0.674/0.723
	>45 (n = 6/6)	56.97 (37.54–79.36)	71.93 (61.46–81.54)	0.109/0.134
LH/FSH ratio, median	16–30 (n = 6/6)	1.55 (0.78–7.83)	1.36 (0.76–2.92)	0.749/0.820
	31–45 (n = 8/8)	1.37 (0.63–3.01)	0.97 (0.43–1.84)	0.227/0.246
	>45 (n = 6/6)	0.45 (0.21–0.55)	0.37 (0.25–0.73)	0.688/0.725
E2 (pmol/L), median	16–30 (n = 6/6)	250.50 (91.6–784.9)	360.55 (114–1168.3)	0.262/0.314
	31–45 (n = 8/8)	421.80 (132.4–515.5)	363.50 (260–976.2)	0.674/0.718
	>45 (n = 6/6)	48.45 (34–72.1)	35.00 (34–37)	**0.016**/**0.029**
PRL (ng/mL), median	16–30 (n = 6/6)	15.77 (4.85–21.22)	8.71 (6.9–23.89)	0.337/0.394
	31–45 (n = 8/8)	12.08 (5.3–31.55)	12.62 (7.98–25.45)	0.753/0.806
	>45 (n = 6/6)	6.32 (4.5–13.08)	8.16 (4.45–13.03)	0.873/0.936
TES (ng/mL), median	16–30 (n = 6/6)	0.47 (0.36–0.82)	0.63 (0.36–0.77)	0.810/0.856
	31–45 (n = 8/8)	0.32 (0.21–0.43)	0.32 (0.17–0.58)	0.958/0.982
	>45 (n = 6/6)	0.29 (0.2–0.41)	0.27 (0.23–0.6)	0.747/0.792
DHEAS (µg/dL), median	16–30 (n = 6/6)	313.0 (206–413)	313.0 (231–474)	0.873/0.939
	31–45 (n = 8/8)	152.00 (82–257)	124.50 (84–487)	0.753/0.797
	>45 (n = 6/6)	66.50 (29–119)	116.0 (44–225)	0.109/0.131
PRG (ng/mL), median	16–30 (n = 6/6)	0.60 (0.3–0.9)	1.60 (0.3–18.7)	0.463/0.498
	31–45 (n = 8/8)	3.50 (0.2–13.78)	3.00 (0.1–15)	0.958/0.984
	>45 (n = 6/6)	0.10 (0.1–0.4)	0.10 (0.1–0.2)	0.902/1.00

Abbreviations: LH, luteinizing hormone; FSH, follicle-stimulating hormone; E2, estradiol; PRL, prolactin; TES, testosterone; DHEAS, dehydroepiandrosterone sulfate; PRG, progesterone; KC, Keratoconus; HC, Healthy Controls. Values in bold indicate statistical significance.

**Table 6:** Hormonal levels in KC based on disease progression.

Hormone	Group	Median	*p*-Value/Monte Carlo *p*-Value
LH (mIU/mL)	Non-Progression (n = 63)	3.55 (0.97–55.78)	0.509/0.515
	Progression (n = 14)	3.03 (0.91–31.69)	
FSH (mIU/mL)	Non-Progression (n = 63)	3.23 (0.71–79.36)	0.424/0.438
	Progression (n = 14)	3.90 (1.56–58.11)	
LH/FSH ratio	Non-Progression (n = 63)	0.90 (0.21–4.20)	0.161/0.154
	Progression (n = 14)	0.74 (0.31–1.62)	
E2 (pmol/L)	Non-Progression (n = 63)	113.30 (34–784.9)	0.476/0.479
	Progression (n = 14)	104.95 (59.9–212.3)	
PRL (ng/mL)	Non-Progression (n = 63)	8.94 (3.20–31.55)	0.480/0.482
	Progression (n = 14)	10.76 (3.92–21.22)	
TES (ng/mL)	Non-Progression (n = 63)	4.21 (0.2–10.7)	0.312/0.319
	Progression (n = 14)	3.48 (0.21–12.11)	
DHEAS (µg/dL)	Non-Progression (n = 62)	219.50 (25–736)	0.128/0.132
	Progression (n = 14)	312.00 (71–747)	
PRG (ng/mL)	Non-Progression (n = 63)	0.20 (0.1–8.3)	0.846/0.848
	Progression (n = 14)	0.20 (0.1–0.5)	

Abbreviations: LH, luteinizing hormone; FSH, follicle-stimulating hormone; E2, estradiol; PRL, prolactin; TES, testosterone; DHEAS, dehydroepiandrosterone sulfate; PRG, progesterone; KC, Keratoconus. Values in bold indicate statistical significance.

**Table 7:** Hormonal levels in KC based on treatment method.

Hormone	Group	Median	*p*-Value/Monte Carlo *p*-Value	Post Hoc *p*-Value		
				CXL-No Surgic.	CXL-PK	No Surgic. -PK
LH (mIU/mL)	No surgical. (n = 67)	3.55 (0.91–55.780)	0.570/0.580	-	-	-
	CXL (n = 11)	3.11 (1.42–11.97)				
	PK (n = 26)	3.74 (1.31–8.35)				
FSH (mIU/mL)	No surgical. (n = 67)	3.68 (0.56–55.78)	**0.035**/**0.031**	0.168	**0.045**	0.551
	CXL (n = 11)	3.17 (0.71–8.83)				
	PK (n = 26)	7.12 (1.38–17.97)				
LH/FSH ratio	No surgical. (n = 67)	0.85 (0.21–9.54)	**0.017**/**0.013**	0.159	**0.014**	0.275
	CXL (n = 11)	1.03 (0.37–4.20)				
	PK (n = 26)	0.51 (0.31–1.52)				
E2 (pmol/L)	No surgical. (n = 67)	105.80 (34–784.90)	0.350/0.353	-	-	-
	CXL (n = 11)	115.35 (36–483)				
	PK (n = 26)	101.80 (59.9–146.8)				
PRL (ng/mL)	No surgical. (n = 67)	10.14 (3.2–51.78)	0.079/0.080	-	-	-
	CXL (n = 11)	10.19 (4.1–26.37)				
	PK (n = 26)	6.49 (3.92–13.95)				
TES (ng/mL)	No surgical. (n = 67)	3.39 (0.2–10.7)	0.159/0.159	-	-	-
	CXL (n = 11)	4.33 (0.21–12.11)				
	PK (n = 26)	4.98 (2.3–8.49)				
DHEAS (µg/dL)	No surgical. (n = 67)	230.00 (25–747)	**0.023**/**0.022**	0.472	**0.029**	0.128
	CXL (n = 11)	286.00 (103–736)				
	PK (n = 25)	174.00 (79–336)				
PRG (ng/mL)	No surgical. (n = 67)	0.20 (0.1–13.8)	**0.003**/**0.002**	0.132	**0.003**	0.075
	CXL (n = 11)	0.20 (0.1–8.3)				
	PK (n = 26)	0.10 (0.1–0.6)				

Abbreviations: LH, luteinizing hormone; FSH, follicle-stimulating hormone; E2, estradiol; PRL, prolactin; TES, testosterone; DHEAS, dehydroepiandrosterone sulfate; PRG, progesterone; CXL, corneal cross linking; PK, penetrating keratoplasty. Values in bold indicate statistical significance.

**Table 8:** Associations between Pentacam/ORA values and Hormone levels in men.

Hormone	Reported Statistic	Kmax	TCT	CCT	Q-Val(f)	Q-Val(b)	Elev(f)	Elev(b)	CH	CRF
LH (n = 135)	Spearman’s rho	0.111	0.015	0.010	−0.095	−0.095	0.066	0.023	0.162	0.091
	*p*-value	0.202	0.867	0.907	0.272	0.272	0.447	0.795	0.061	0.295
FSH (n = 135)	Spearman’s rho	0.081	0.009	0.007	−0.097	−0.114	0.018	−0.003	0.103	0.007
	*p*-value	0.349	0.920	0.937	0.264	0.189	0.840	0.972	0.234	0.933
LH/FSH ratio (n = 135)	Spearman’s rho	−0.036	0.018	0.015	0.068	0.078	0.004	−0.001	0.019	0.062
	*p*-value	0.681	0.838	0.865	0.432	0.368	0.962	0.994	0.831	0.477
E2 (n = 135)	Spearman’s rho	0.062	−0.099	−0.099	−0.012	−0.020	0.130	0.091	−0.042	−0.142
	*p*-value	0.472	0.251	0.254	0.889	0.815	0.131	0.295	0.628	0.101
PRL (n = 135)	Spearman’s rho	**0.185**	−0.091	−0.118	**−0.223**	−0.147	0.128	0.039	0.036	−0.094
	*p*-value	**0.032**	0.294	0.174	**0.009**	0.089	0.140	0.650	0.680	0.277
TES (n = 135)	Spearman’s rho	**−0.188**	**0.247**	**0.248**	0.108	0.145	**−0.175**	**−0.185**	**0.218**	0.167
	*p*-value	**0.029**	**0.004**	**0.004**	0.211	0.094	**0.042**	**0.032**	**0.011**	0.053
DHEAS (n = 134)	Spearman’s rho	−0.032	−0.131	−0.157	−0.098	−0.018	−0.020	−0.019	−0.021	−0.045
	*p*-value	0.713	0.131	0.070	0.258	0.835	0.819	0.825	0.810	0.603
PRG (n = 135)	Spearman’s rho	0.088	**−0.196**	**−0.198**	−0.085	−0.092	0.103	0.106	0.028	0.005
	*p*-value	0.312	**0.023**	**0.022**	0.326	0.288	0.232	0.221	0.744	0.958

Abbreviations: LH, luteinizing hormone; FSH, follicle-stimulating hormone; E2, estradiol; PRL, prolactin; TES, testosterone; DHEAS, dehydroepiandrosterone sulfate; PRG, progesterone; TCT, thinnest corneal thickness; CCT, central corneal thickness; CH, corneal hysteresis; CRF, corneal resistance factor. Values in bold indicate statistical significance.

**Table 9:** Linear Regression Analysis of Hormonal Predictors of Pentacam and ORA Parameters.

Hormone	Reported Statistic	Kmax	TCT	CCT	Q-Val(f)	Q-Val(b)	Elev(f)	Elev(b)	CH	CRF
LH (n = 174)	*p*-value	0.610	0.889	0.847	0.453	0.915	0.907	0.793	**0.036**	**0.016**
FSH (n = 174)	*p*-value	0.237	0.582	0.721	0.240	0.732	0.192	0.218	0.408	0.354
LH/FSH ratio (n = 174)	*p*-value	0.534	0.735	0.790	0.430	0.555	0.462	0.599	0.813	0.922
E2 (n = 174)	*p*-value	0.286	0.457	0.497	0.271	0.559	0.892	0.573	0.143	0.150
PRL (n = 174)	*p*-value	0.266	0.705	0.512	0.145	0.665	0.680	0.992	0.512	0.838
TES (n = 174)	*p*-value	0.372	0.630	0.361	0.860	0.768	0.223	0.517	0.679	0.694
DHEAS (n = 173)	*p*-value	0.326	0.611	0.429	0.471	0.877	0.901	0.541	0.477	0.110
PRG (n = 174)	*p*-value	0.535	0.487	0.374	0.420	0.959	0.506	0.705	0.906	0.990

Abbreviations: LH, luteinizing hormone; FSH, follicle-stimulating hormone; E2, estradiol; PRL, prolactin; TES, testosterone; DHEAS, dehydroepiandrosterone sulfate; PRG, progesterone; TCT, thinnest corneal thickness; CCT, central corneal thickness; CH, corneal hysteresis; CRF, corneal resistance factor. Values in bold indicate statistical significance.

**Table 10:** Hormonal levels in KC based on Kmax.

Hormone	Group	Median	*p*-Value/Monte Carlo *p*-Value
LH (mIU/mL)	Kmax < 49 D (n = 9)	2.98 (1.68–5.09)	0.174/0.170
	Kmax 49–53 (n = 10)	5.11 (1.42–30.19)	
	Kmax 53.1–58 (n = 28)	3.51 (0.91–55.78)	
	Kmax ≥ 58 D (n = 57)	3.24 (0.95–31.69)	
FSH (mIU/mL)	Kmax < 49 D (n = 9)	2.31 (0.71–8.83)	0.066/0.059
	Kmax 49–53 (n = 10)	6.02 (0.56–55.83)	
	Kmax 53.1–58 (n = 28)	4.11 (1.33–79.36)	
	Kmax ≥ 58 D (n = 57)	3.53 (1.23–58.11)	
LH/FSH ratio	Kmax < 49D (n = 9)	1.19 (0.37–4.2)	0.241/0.245
	Kmax 49–53 (n = 10)	0.48 (0.29–9.54)	
	Kmax 53.1–58 (n = 28)	0.85 (0.21–2.83)	
	Kmax ≥ 58D (n = 57)	0.83 (0.31–3.01)	
E2 (pmol/L)	Kmax < 49 D (n = 9)	113.70 (70.3–333.80)	0.897/0.903
	Kmax 49–53 (n = 10)	100.55 (36–232)	
	Kmax 53.1–58 (n = 28)	118.75 (34–784.9)	
	Kmax ≥ 58 D (n = 57)	101.80 (36–515.5)	
PRL (ng/mL)	Kmax < 49 D (n = 9)	8.22 (5.74–17.27)	0.807/0.811
	Kmax 49–53 (n = 10)	7.94 (5.86–14.07)	
	Kmax 53.1–58 (n = 28)	9.26 (3.92–51.78)	
	Kmax ≥ 58 D (n = 57)	10.44 (3.2–31.55)	
TES (ng/mL)	Kmax < 49 D (n = 9)	4.86 (0.46–8.72)	0.328/0.332
	Kmax 49–53 (n = 10)	4.51 (0.25–7.20)	
	Kmax 53.1–58 (n = 28)	3.27 (0.2–7.61)	
	Kmax ≥ 58D (n = 57)	4.01 (0.29–12.11)	
DHEAS (µg/dL)	Kmax < 49 D (n = 9)	315.00 (186–736)	0.087/0.087
	Kmax 49–53 (n = 10)	184.50 (62–486)	
	Kmax 53.1–58 (n = 28)	215.00 (29–525)	
	Kmax ≥ 58 D (n = 56)	262.50 (25–747)	
PRG (ng/mL)	Kmax < 49 D (n = 9)	0.30 (0.2–7.0)	0.079/0.075
	Kmax 49–53 (n = 10)	0.20 (0.1–8.3)	
	Kmax 53.1–58 (n = 28)	0.20 (0.1–6.6)	
	Kmax ≥ 58 D (n = 57)	0.20 (0.1–13.8)	

Abbreviations: LH, luteinizing hormone; FSH, follicle-stimulating hormone; E2, estradiol; PRL, prolactin; TES, testosterone; DHEAS, dehydroepiandrosterone sulfate; PRG, progesterone; Kmax, Maximum Simulated Keratometry.

## Data Availability

The raw data supporting the conclusions of this article will be made available by the authors on request.

## Abbreviations

The following abbreviations are used in this manuscript:

KC	Keratoconus
HC	Healthy controls
ECD	Ectatic Corneal Diseases
PMD	Pellucid Marginal Degeneration
CXL	Corneal Cross-Linking
PK	Penetrating keratoplasty
Kmax	Maximum Simulated Keratometry
CCtmin	Minimum Central Corneal Thickness
Q-val(f)	Q-value front
Q-val(b)	Q-value back
Elev(f)	Elevation front
Elev(b)	Elevation back
CH	Corneal hysteresis
CRF	Corneal resistance factor
BAD-D	Belin/Ambrosio Ectasia display
PRL	Prolactin
PRG	Progesterone
LH	Luteinizing Hormone
FSH	Follicle-Stimulating Hormone
DHEAS	Dehydroepiandrosterone Sulfate
E1	Estrone
E2	17-β Estradiol
E3	Estriol
TES	Testosterone
ORA	Ocular Response Analyzer
MMPs	Matrix Metalloproteinases
ECM	Extracellular Matrix
HCFs	Healthy Corneal Stromal Fibroblasts
HKCs	Keratoconus Corneal Stromal Cells
IL-6	Interleukin 6
IL-8	Interleukin 8
PIP	Prolactin-induced protein
